# White Matter Hyperintensities Predict Driving Behavior Changes in Preclinical Alzheimer’s Disease

**DOI:** 10.1002/alz70862_110306

**Published:** 2025-12-23

**Authors:** Chia‐Ling Phuah, Madhur Parihar, Yasheng Chen, David B. Carr, Jin‐Moo Lee, Ganesh M. Babulal

**Affiliations:** ^1^ Dignity Health dba Barrow Neurological Institute/St. Joseph's Hospital & Medical Center, Phoenix, AZ USA; ^2^ Washington University School of Medicine in St. Louis, St. Louis, MO USA; ^3^ Washington University School of Medicine, St. Louis, MO USA; ^4^ Washington University School of Medicine, Saint Louis, MO USA; ^5^ University of Johannesburg, Johannesburg, Gauteng Province South Africa

## Abstract

**Background:**

White matter hyperintensities (WMH) compromise cognitive reserve, potentially accelerating dementia onset in etiologies like Alzheimer’s disease (AD). Extant neuroimaging studies link WMH severity to driving cessation in older adults (OA). Region‐specific WMH distributions reflect distinct etiologies, offering insights into differential cognitive and functional impacts. We investigated WMH impact on complex cognitive performance through longitudinal analysis of naturalistic driving behavior in OA. Our investigation bridges gaps in understanding how subtle structural changes influence real‐world cognitive functioning.

**Method:**

We analyzed data from 212 cognitively intact OA (aged ≤65 years, CDR=0) in the DRIVES (Driving Real‐World In‐Vehicle Evaluation System) Project cohort, with 3T MRI brain scans within two years of starting longitudinal driving assessments. We examined 16 driving metrics aggregated monthly using in‐vehicle data loggers, encompassing trip characteristics, speed/acceleration/braking patterns, and route complexity. We quantified WMH using a deep learning algorithm, enabling precise measurements of total WMH volume and region‐specific WMH distributions. Linear mixed‐effects models with random coefficients, adjusted for demographic factors (age, sex, race, education) and socioeconomic status (area deprivation index), assessed WMH influence on longitudinal changes in driving performance. Significance was set at FDR‐adjusted *p*<0.05.

**Result:**

Our study included 74,275 weeks of driving data (2015‐2024, average follow‐up 6.1 years). Increased WMH burden correlated with decreased trip frequency (*p* = 0.0005), fewer near‐home trips (*p* = 0.0004), reduced unique destinations (*p* = 0.0003), and lower driving entropy (*p* = 0.001) over time. Decrease in driving complexity was primarily driven by posteriorly‐located WMH lesions, especially in parietal and occipital regions (β=‐0.09, *p* = 0.002 and β=‐0.10, *p* = 0.0009, respectively) involved in visual processing, motion detection and spatial awareness. WMH impact on driving behavior intensified over time in participants developing cognitive impairment (*n* = 36), manifesting as increased hard breaking and impact events.

**Conclusion:**

WMH in OA significantly impacts driving behavior, leading to latent self‐regulation and reduced driving complexity. Cognitive impairment with WMH increases risky driving. Posterior WMH influence suggests a dominant role of AD pathology in driving performance decline. WMH shows potential as a biomarker for identifying individuals at higher risk of unsafe driving and premature driving cessation, highlighting its value in early screening and intervention strategies for road safety among aging populations, particularly those at risk for AD.

